# The Genesis of the Contour Filling

**Published:** 1891-08

**Authors:** George S. Allan

**Affiliations:** New York City


### THE GENESIS OF THE CONTOUR FILLING.[1]

BY GEORGE S. ALLAN, D.D.S., NEW YORK CITY.

So generally accepted is it by the profession at large that the contour filling represents the highest development of the art in conservative dentistry, that it is as much as a man's reputation is worth to say a good word for the old-fashioned face filling of years gone by. Practice and precept, it is true, do not always follow the same road; still it is a healthy sign of growth to see the highest ideal kept well in the foreground in all our discussions and writings. No subject has been so well and so ably handled, by the wise men in our profession, as the one that presses the claims and advantages of contour work. So true is this, that he who would start on the hunt for one single brand-new thought or idea on the subject would have a weary road to travel and have little to show for his time and trouble when he came to foot up his returns.

At the best he could only hope to attract attention by giving some more beautiful or apt illustration to some well-worn idea or principle that had already become the common property of all. So I turn aside from this uninviting field of labor to another,—one more practical, and I hope, therefore, more interesting, and will confine my thoughts entirely within the lines marked out by the title of my paper,—the genesis of the contour filling, or how it is made.

And this I do for the further reason that, so far as I know, the cardinal rules to be kept in mind in this kind of work have never been presented to the profession in short compass.

Even within these lines, I doubt much whether I can give you much that is new. Still, I think a little less attention has been given to practical instruction in building up the contour filling than to preaching about its many advantages and beauties. This close attention to one thought will also preclude entering into any discussion as to when and where to attempt the full restoration in the shape of the tooth under treatment. Individual judgment largely controls practice in all cases, and the wise dentist, like the wise physician, always adopts the elective motto. The wisest short law I have ever seen pertaining to this point came to me in a

---

[1] Read before the Section of Dental and Oral Surgery of the American Medical Association held in Washington, D. C., May, 1891.

private letter from an acknowledged authority on the subject, and sums as follows:

"To understand me more completely, I would state that I have not for a considerable time been in favor of inserting very large gold fillings in this manner, and for the reasons you have given in your paper, that the structure and elements of the tooth are not such as to promise a durable retention of the mass of gold by the weakened tooth. But for medium-sized and small cavities, I am equally convinced, from long experience and observation of my own, and the work of others, that no other method offers the same degree of permanency or usefulness."

By so doing, he placed himself in the position of the philosopher who said he always obtained what he wished for, and added, *sotto voce*, "I take good care never to wish for what I cannot obtain."

Taking up, then, the building or making of a contour filling, a threefold division of the subject naturally presents itself,—

1. The preparing of the cavity.
2. The placing therein of the filling material.
3. Finishing and polishing the completed work.

Now a good deal that may be said about the preparing of the cavity will apply to the simpler and easier operations of face and crown fillings; but this one wide difference must be constantly kept in mind,—viz., as the size of the cavity increases, the difficulties and dangers increase, not proportionately, but in a geometrical ratio, and therefore a relatively greater care and attention must be given to all the details of the larger and more complex operation. A few quick, sharp cuts will suffice for preparing a simple crown cavity, but they would fall far short of filling the bill in any case that we propose to consider this evening.

As the architect or engineer sees his completed work before the actual is even started, so should the dentist be able to discern the full size, shape, and figure of his proposed restoration, and each step should be so carefully planned and made to fit the next that, in the completed whole, nothing may be wanting.

Very much of success depends on bearing this safe rule in mind. In fact, it is difficult to conceive how one can plan wisely unless he does. On the size and shape of the cavity, on the proper distribution of retaining undercuts or pits, so as to protect and strengthen weak walls and throw the burden on strong ones, much good judgment can be placed, and the writer is convinced that just here may be found the cause of many failures. Undercuts are made too deep, and retaining-pits made in such positions or manner as to either weaken the tooth or endanger the pulp. Deep undercuts, though they make operations easier, are seldom called for, and endanger the completed work, and this they do in two ways,—first, by weakening the walls of the cavity, and secondly, by making real obstacles to forming a homogeneous, well-packed filling.

The deep undercut, though it holds the great bulk of the gold in place, is itself difficult to fill. To do so well takes much time and care and the use of exceedingly small pluggers, and this they may not receive. Where all the walls of a cavity are standing, and the face of the tooth adjacent to the cavity is perfect, the walls of the cavity should be left as nearly parallel as possible, and no pits of any kind made. In fact, nearly parallel walls should be the rule, and pits always avoided, when possible.

The real study and judgment is called for in those cases where the natural face or faces of the tooth have been lost, either by decay or the too free use of the file and chisel, for, as will be referred to a little later on in speaking of packing the gold, a point of considerable interest and value arises in these cases, as to whether the filling should be allowed to overlap the walls of the cavity, and simply lie against the face of the tooth on the outside, or shall be made continuous with the walls of the cavity, and bulge only from the cavity itself.

Believing fully, as I do, that overlapping gold is gold in a dangerous position, I would strongly advocate such a preparation of the cavity as will minimize this danger, even if, by so doing, the full realization of our ideal in contouring be not carried out.

It is a sort of belief with many that, in all cases, the packing of the gold should be commenced at the cervical wall or base of the cavity. This is a mistake. Oftentimes more certain and rapid work can be done by starting the filling back in the grinding surface, and building downward. And so this point should be considered, and the cavity shaped accordingly.

As the enamel forms the edges of most large cavities, its proper management is a point of interest. Long ago I advocated, in a paper read before the New York Odontological Society, the complete removal of the thin edge of enamel often found at the neck of the tooth; and this I did for the double reason that it was only slightly adherent to the dentine just there, and so liable to split off during the operation of filling, and, secondly, it was very difficult to make a smooth edge on it.

Since that date I have seen no reason to alter my judgment in respect to this method of practice, but have had many confirmatory ones brought to my notice. Leaving the thin edge leaves a weak spot, and that is bad.

A final point to observe in shaping the cavity consists in making the edges smooth and polished, and just here the great advantage of the dental engine, with its rapidly revolving bur, comes to the front. No hand instrument, no matter how much care is used, can compete with it. A sharp, well-cut bur will do in a few minutes far more effective and perfect work than the sharpest hand-instrument can in a far longer time. If, in addition to the bur, the edges are polished with the wood point armed with powder, or still better, with an uncut round, soft-iron point, armed with diamond dust, perfect edges can be quickly obtained.

We come now to what many will think the most important part of the work, and the one in connection with which probably more science and skill can be employed than in any other. Two great essentials are to be here considered,—the perfect adaptation of the gold to all the walls of the cavity, and the accomplishing this with a minimum amount of force; and a third may be added,—that the filling be made homogeneous and solid throughout.

Imperfect adaptation makes failure almost a certainty, and undue force (and by this I mean any amount of force over and above that required to condense the gold) is almost equally fatal in the end. How, then, first of all, shall we proceed to make the gold fit the cavity?

The quality in gold that we make use of in building up a filling of this character—viz., its welding property when pure and freshly annealed—is not one that can be trifled with. It is our servant if we handle it rightly; our master, if we slight it in any particular.

In skilful hands pure gold is almost as pliable and obedient to the touch as the clay the sculptor uses to fashion the child of his fancy, but there is this difference: the clay can be worked over and over again,—a little added here, and as much taken off there,—and so long as it is kept moist, it responds to the brain back of it; but not so with gold. Place a bit in position in such a manner as to insure perfect contact with that already in place, and the union will be perfect, and it becomes a part of it; but you cannot try a second time; the first is the only one that will be allowed you; if it does not take its proper position at once, you may be sure that something is wrong, and you cannot make that wrong right by using extra force. The moment that it is done, the evil that resides in the metal and that heretofore has been dormant, manifests itself. It becomes stubborn, brittle, and cranky, and will not do anything you want it to do, but will persist in doing everything you most object to,—like some individuals, it has a dual nature, and we must beware how we call out the perverse side.

Then, again, the amount of force used must always be proportionate to the size of the pledget to be packed, and exactly, too; not enough force fails to insure solidity, and too much has a tendency to bring out the harsh qualities alluded to. As the pledget must be packed in the exact position in which it is first placed, and cannot be moved from it, great care and good judgment are required to avoid pits, and to make certain that the surface is kept even, for it is much more difficult to fill a pit or sharp depression than to continue a flat surface, and the permitting of pits near the walls of the cavity is especially to be avoided, for the extra force necessary to fill them too often weakens the walls or even crumbles the enamel. So the rule to be observed is to, as far as possible, carry a uniform surface upward, and as the filling grows keep the marginal portions a little in advance of the centre.

Another advantage that results from this plan of procedure is that it makes more rapid work possible, in that you can use larger points. A pit or depression near the walls necessitates the employment of small points, for the point in use must always be a trifle smaller than the pit to be filled. This suggestion is by no means an unimportant one, and a little thought will soon convince one of that fact. The correct packing of gold is, in truth, an art of itself, and requires an educated touch and correct eye.

The celebrated painter, Meissonier, always took the greatest care of his hands, keeping them scrupulously clean, and even tender, by the constant use of gloves. He said that a painter's touch must be most sensitive and delicate. So it should be with the dentist, and he ought to be just as careful and particular. Long finger-nails or callous finger-tips are to be avoided.

Where to start a filling is a matter of some moment. It is generally commenced at the cervical wall, but cases frequently occur where the commencement may be made with advantage back on the grinding face of the tooth, and the filling carried backward or forward to the floor of the cavity. An advantage frequently arises from this method, for, by carrying the gold along the sides of the cavity towards the bottom, any movement of the gold is avoided, and pits to accomplish this same purpose are not required. Any one who has not tried this plan will be astonished to find how frequently it facilitates the operation. Deep pits, in the writer's opinion, are great nuisances, and are only admissible when other means fail to accomplish their purpose.

What has been said regarding the manner of making a filling leads naturally to a consideration of the points to be used, their shape, and the best methods of applying the force necessary for condensation of the gold. If the gold is to be built up layer by layer, in a series of planes, the points themselves, in fact, should have flat surfaces, and this will be found to be a correct statement. The late Dr. Varney was the first to incorporate this principle in a series of pluggers especially intended for packing cohesive gold, and the set made from patterns furnished by him to this day stand unequalled for their special adaptation for contour work. The points all have plain surfaces, and are very finely and evenly serrated. Slight modifications only have been made in them since his day, so completely did he work out his theory in steel.

A round point may have some limited utility at times, but never can be relied upon to any extent. It would be a matter of the greatest difficulty to make a large, uniformly-packed filling by their use only. Deep serrations are faulty in that they cut the gold and require extra power to force the gold into a solid.

How to apply the requisite pressure is now to be considered, and possibly it is the most important consideration of all. How to apply just enough force for the purpose, and to apply it quickly and uniformly and evenly. How to apply the "quantum sufficit" and no more, and in a manner to give your patient the least discomfort and lighten the labor of the dentist, is a serious problem.

I take it that few would seriously consider hand-pressure alone as offering the best solution. Let alone its being the most laborious and tedious method, it rarely produces perfect work. We cannot get along without it, especially in commencing operations; but good judgment and good work alike demand that it be supplemented by some means more under control, more direct in its action, and developing more power; and this can only be done by resorting to some one or more of the various devices which the ingenuity and thought of the profession has placed in our hands for utilizing the power of the momentum, or, in plainer terms, the mallet.

The late Dr. Atkinson, whose memory we hold in respect, and whose loss we deplore, was probably the first to suggest this means of obtaining the desired end, and in his hands, and in the hands of his followers, the hand-mallet was made to do most excellent service; but it was soon found that it offered only a partial solution of the problem; to mallet for one's self was awkward and oftentimes impracticable, and the impossibility of making two brains work in harmony made the assistant malleter as often a nuisance as a help; and careful operators soon gave it up as being impracticable. The automatic mallet, in some of its various forms, took its place, and so well did it do its work that it will probably always hold its own and retain a well-deserved place in the dentists outfit. Then followed the electric mallet, a step in advance, and a big one; but it had many inherent defects that greatly impeded its general adoption.

To-day the mechanical mallet is slowly but surely coming to the front, and the writer feels certain that the day is not far distant when it, in some of its modifications, will supersede all others, and for reasons that will now be stated as clearly as possible.

Let it be premised that the more closely the force required can be made to simulate hand-pressure the better it will be in all ways, —safer for the tooth and easier for the patient to bear. Now, this is just what the mechanical mallet does,—in truth, it is pressure intermittently applied, and in nowise is to be likened to the hammer-like blow that is given either by the hand-mallet, the automatic, or the electric.

Take a look at the mechanical mallet in operation and notice how it works. You will see that the point is placed in contact with the gold and gently pushed forward. This throws the farther end of the mandrel holding the point back, so that the lug or rounded bit of steel, with which the rapidly revolving wheel is armed, comes in contact with it, and it is pushed forward, and this is repeated with every revolution of the wheel, so that from one thousand to three thousand impulses may be given to the point every minute, the direction, number and power of these impulses being perfectly under the control of the operator.

In the mallet I employ, which is the Bonwill mallet, as modified by Dr. S. Perry and Mr. Weber, there are eighty threads to the inch in the adjusting screw, and forty notches in the collar, so that a movement of the collar one notch brings the plugger mandrel one thirty-two-hundredth of an inch, or the thickness of the diameter of a human-blood corpuscle, nearer to or father away from the revolving wheel, and yet small as this distance is, it is distinctly appreciable to the operator and patient alike. Does it not look reasonable that a forward push movement of the point, through these small distances, must be comparatively safe and can be made to expend itself in the packing of the gold, and the packing only?

A valuable point I have observed is that where the serrations of the point are rightly made,—that is, having one side longer than the other,—the point travels over the surface of the filling and has simply to be guided by the operator, so that if placed near the centre of the filling, it will move over the face of the gold in just the direction and manner required; thus it becomes the easiest thing in the world to pack towards the walls of the cavity. The gold is plastered in position, as it were, easily and rapidly.

The kind and quality of the push can be regulated in several ways other than by the adjustment collar,—by increasing or diminishing the speed of the motor, a great change is at one perceptible, and again, the educated touch will hold the point against the gold so as to insure perfect packing, and no force wasted. In fact, through a wide variation of power, it is under perfect control.

A few words, in closing, on the finishing. This is a matter of detail, and often of sad neglect. Too high a polish cannot be given to the perfected work. Do the best we can, and we will fall short, far short, of Nature's model. Time and labor cannot be thrown away, if intelligently employed in the finish.

I mentioned, in speaking of packing gold, that solidity and homogeneity were essential considerations. I grant that a filling that is hard enough to resist pressure, and is perfectly adapted to the walls of the cavity, will prove effective; but perfect adaptation and the requisite hardness, as a rule, also mean homogeneity; not always, though ; yet I feel certain of my position when I say that it is not only far easier to finish a uniformly dense filling, but that the work can be done in a shorter time, and always in a more satisfactory manner. I know of no more discouraging labor than that employed in attempting to put a finished surface on an imperfectly packed filling.

Where a tooth has been mutilated by disease, or the file and disk, a question often arises, as before mentioned, as to whether the gold shall be allowed to overlap the edges of the cavity, or simply be rounded out from them. It is doubtful whether gold can be made to lay against tooth-substance in such a manner as to prevent the ingress of fluids. The thinner and wider the overlapping gold, or the thinner and deeper the overlapping gold, the greater the doubt and uncertainty. If the diameter of the gold is one-third or one-half greater than that of the cavity, we may be almost certain of trouble in the near future.

The difficulty in making a perfect edge is also greatly increased in these cases, and so it is a safe rule to observe to finish the gold to fit the cavity, and to take the place of an overlapping lid as little as possible.

In shaping, the articulation should be always left in such a condition that the filling should, in mastication, be pressed back into the tooth, not out from the cavity.

As a means of education, I know of nothing superior to making fillings out of the mouth. Any one who has not tried it will be surprised at the amount of instruction that can be acquired in this way, and in no other. Any earnest worker who makes the experiment will be astonished to find, not only that the operation is not easy under these simple conditions, but as he aims for perfection, how difficult it is to attain. His edges will remain imperfect, and his powders will scratch, and he will wonder how he ever succeeds in the mouth.

To sum up: In preparing your cavity, be careful to avoid deep undercuts or pits, and throw as little strain on the enamel as possible, and make clean, polished edges. In packing the gold, bear in mind that your filling must be homogeneous throughout. Perfectly fill the cavity and build up in a series of planes, and in finishing imitate nature in the high polish you put upon your work, for, do your best, you will fall far short of her beautiful handiwork. To do perfect contour work requires care and skill. With practice and experience one will be surprised to find how often the impossible becomes possible and the difficult easy of attainment.

